# Developing a calibration method to utilize low‐dose chest CT for assessment of coronary artery calcification score

**DOI:** 10.1002/acm2.70614

**Published:** 2026-05-12

**Authors:** Kuei‐Yuan Hou, Ching‐Ching Yang

**Affiliations:** ^1^ Department of Radiology Cathay General Hospital Taipei Taiwan; ^2^ Department of Medical Imaging and Radiological Sciences Chung‐Shan Medical University Taichung Taiwan; ^3^ Department of Medical Imaging and Radiological Sciences Kaohsiung Medical University Kaohsiung Taiwan; ^4^ Department of Medical Research Kaohsiung Medical University Hospital Kaohsiung Taiwan

**Keywords:** coronary artery calcium scores, ECG‐gated calcium scoring CT, low‐doses chest CT

## Abstract

**Purpose:**

This study aimed to develop a calibration approach for low‐dose CT (LDCT) used in lung cancer screening to enable coronary artery calcium (CAC) scores that reflect those obtained from conventional electrocardiogram (ECG)‐gated calcium scoring CT (CSCT).

**Methods:**

An electron density phantom‐based calibration curve was developed to align Hounsfield unit (HU) values measured on LDCT with those from ECG‐gated CSCT. To evaluate the efficacy of the proposed method, 139 patients undergoing physical examination who received both LDCT and CSCT on the same day were retrospectively enrolled. Agatston and volume scores were quantified for the right coronary artery, left anterior descending artery, left circumflex artery, and left main artery. The Bland‐Altman analysis and two‐sample t‐test were performed to compare CAC scores measured on CSCT and LDCT before and after HU‐based calibration.

**Results:**

Compared with the mean bias observed in Bland‐Altman analysis between CSCT and LDCT, the mean bias between CSCT and calibration LDCT (cLDCT) was substantially reduced. Based on two‐sample t‐test, statistically significant differences in total Agatston scores and total volume scores calculated were observed between CSCT and LDCT for the four coronary arteries. In contrast, no significant differences were found between CSCT and cLDCT. Among patients undergoing diastolic reconstruction in CSCT, the *p*‐values comparing CSCT and cLDCT were 0.94 for the Agatston score and 0.43 for the volume score, whereas corresponding *p*‐values of 0.95 and 0.63 were observed in patients undergoing systolic reconstruction in CSCT.

**Conclusion:**

The proposed calibration approach enables consistent HU mapping across protocols and supports simultaneous assessment of coronary calcium burden and pulmonary pathology on a single LDCT scan.

## 1 INTRODUCTION

As the leading cause of mortality worldwide, coronary artery disease is commonly evaluated using electrocardiogram (ECG)‐gated calcium scoring CT (CSCT) to detect and quantify coronary calcification.^1^, ^2^, ^3^ The Agatston score, a widely used quantitative metric of coronary calcium burden, is typically performed on CSCT acquired at 120 kVp and reconstructed with 3‐mm slice thickness using filtered back projection (FBP).^4^, ^5^ The American Heart Association recommends CSCT with effective dose (ED) ≤ 3.0 mSv.^6^ By comparison, the American College of Radiology recommends low‐dose CT (LDCT) acquired for lung cancer screening with volume CT dose index (CTDI_vol_) ≤ 3 mGy and ED ≤ 1 mSv.^7^ Various strategies have been implemented to reduce radiation exposure in CT.^8^, ^9^, ^10^ Tube current modulation adjusts the mA according to patient body shape, thereby lowering radiation dose compared with fixed‐mA scans.^11^, ^12^ Advanced reconstruction techniques, e.g., iterative reconstruction and deep learning‐based reconstruction, reduce statistical noise associated with low‐dose imaging compared with conventional FBP, allowing for radiation dose reduction while maintaining image quality.^13^, ^14^ Additionally, spectral shaping using vendor‐specific tin or silver filtration can attenuate low‐energy photons to further reduce patient dose.^15^, ^16^ Although the primary purpose of LDCT is lung cancer screening rather than coronary evaluation, the ability to assess coronary artery calcification (CAC) from non‐ECG‐gated chest CT has gained increasing clinical attention.^17^, ^18^, ^19^ However, CAC quantification on LDCT can be significantly influenced by scan parameters and reconstruction techniques, with lower kV scans being more prone to false‐positive results and thicker slices more likely to underestimate CAC. Compared with the Agatston score, total visual scoring and length‐based methods are less affected by Hounsfield unit (HU) variations arising from protocol differences.^20^, ^21^ Although these approaches can provide accurate CAC assessments on non‐ECG‐gated chest CT, they are less practical and not as universally applied as the Agatston score. To enable Agatston scoring on LDCT, a calibration approach that correlates HU values between LDCT and conventional ECG‐gated CSCT is required. In this study, the relationship between HU values obtained from the two scan types was first investigated with electron density (ED) phantoms. Based on this comparison, a HU‐based calibration method was developed for LDCT acquired with silver beam‐shaping filter and deep learning‐based reconstruction, aiming to generate calcium scores consistent with those derived from ECG‐gated CSCT.

## 2 METHODS

### 2.1 Study population

With institutional review board approval, 139 consecutive patients (105 men, 34 women) undergoing physical examination in our hospital were retrospectively enrolled. The mean age was 59.8 ± 10.0 years, mean heart rate (HR) was 65.0 ± 11.1 bpm, and mean body mass index (BMI) was 24.9 ± 3.5 kg/m^2^. The enrolled patients underwent CSCT and LDCT on the same day using a 320‐detector row CT scanner (Aquilion One, Canon Medical Systems, Otawara, Japan) at a single center from November 2024 through August 2025. In CSCT, diastolic reconstruction was performed in 118 patients (84 men and 34 women), while systolic reconstruction was performed in 21 patients (21 men). For patients undergoing diastolic reconstruction, the mean age was 60.0 ± 10.3 years, mean HR was 61.6 ± 7.8 bpm, and mean BMI was 24.7 ± 3.6 kg/m^2^. In patient undergoing systolic reconstruction, the mean age was 58.7 ± 7.8 years, mean HR was 84.0 ± 7.4 bpm, and mean BMI was 26.1 ± 3.3 kg/m^2^.

### 2.2 CSCT scans

CSCT acquisition protocols used in clinical practice were applied in this study. Key parameters are summarized in Table 1. In our department, CSCT scans are performed using a standard copper filter at 120 kVp with automatic tube current modulation (ATCM). The image quality reference of the ATCM system (SureExposure 3D, Canon Medical Systems, Otawara, Japan) is defined as the standard deviation (SD) of HU values in the central region of a homogeneous water phantom. The SD value was set to 30, allowing automatic adjustment of the tube current within a range of 40–500 mA. Images were reconstructed using hybrid iterative reconstruction (Adaptive Iterative Dose Reduction, AIDR) with a soft‐tissue kernel (FC12). For patients with HR below 70 bpm, single‐sector reconstruction was performed at 75% of the R‐R interval, whereas for those with HR above 70 bpm, multi‐sector reconstruction was performed at 40% of the R‐R interval.

**TABLE 1:** Key acquisition parameters for CSCT and LDCT.

	CSCT	LDCT
Acquisition type	Sequenced	Spiral (pitch = 1.388)
Collimation	320 × 0.5 mm	80 × 0.5 mm
Revolution time	0.275 s	0.5 s
Filter material	Aluminum\Copper	Aluminum\Silver
Tube voltage	120 kVp	120 kVp
SD value	30	15
Reconstruction algorithm	AIDR	AiCE
Convolution kernel	FC12	BODY_SHARP
Matrix	512 × 512	512 × 512
Slice thickness	3.0 mm	3.0 mm
Scan field‐of‐view	32 cm	50 cm
CTDI_vol_	4.15 mGy	2.10 mGy

### 2.3 LDCT scans

Routine LDCT acquisition protocols were used in this study. Key parameters are summarized in Table 1. In our department, LDCT scans are performed using a silver beam‐shaping filter at 120 kVp with ATCM. The SD value of the ATCM system (SureExposure 3D, Canon Medical Systems, Otawara, Japan) was set to 15, enabling automatic modulation of the tube current within a range of 40–700 mA. Images were reconstructed using deep learning‐based reconstruction (Advanced intelligent Clear‐IQ Engine, AiCE) with a soft‐tissue kernel (BODY_SHARP).

### 2.4 Energy mapping

**FIGURE 1:**
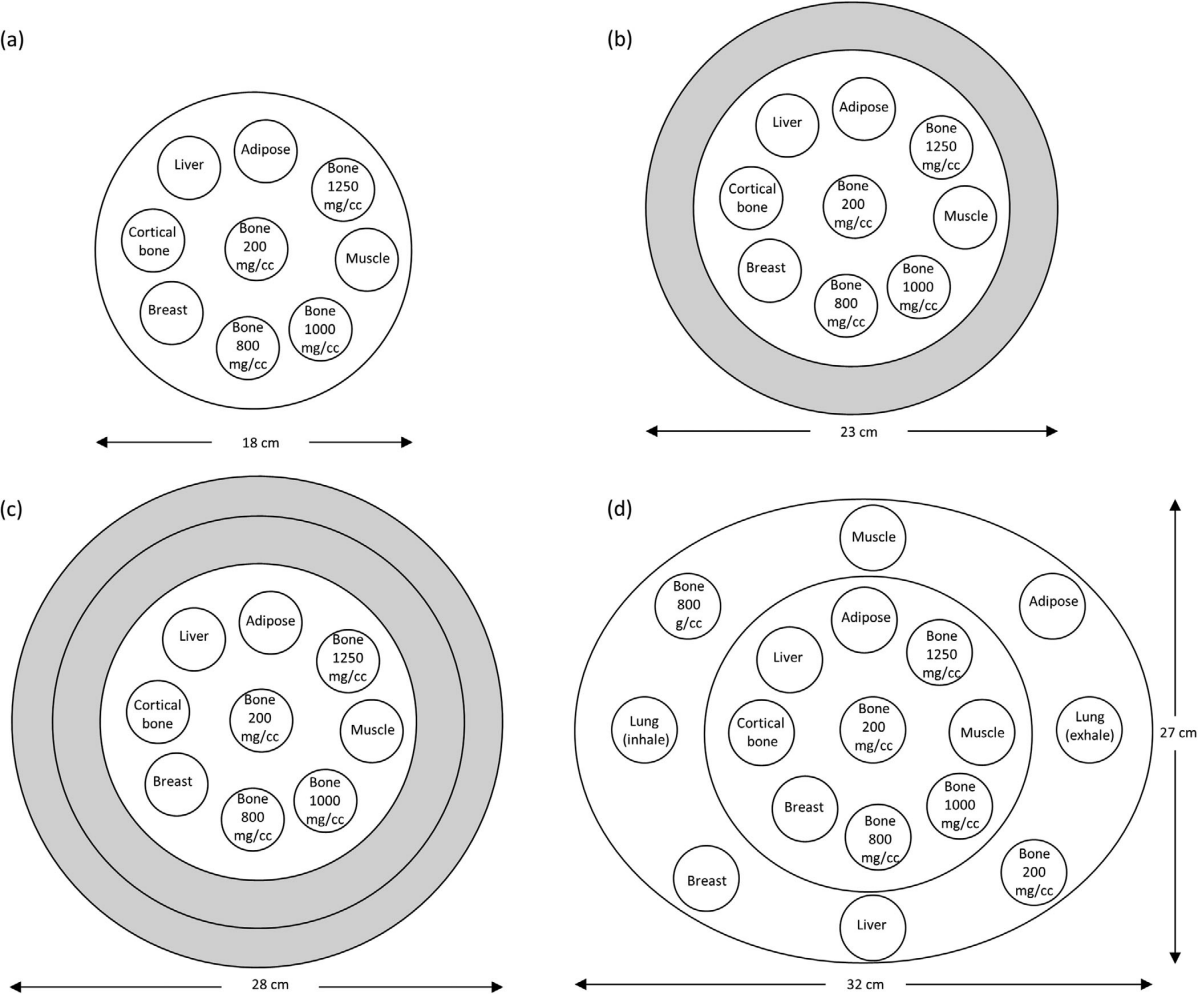
Illustration of electron density (ED) phantoms, including (a) ED1, (b) ED2, (c) ED3, and (d) ED4.

Because different bowtie filters are used for CSCT and LDCT in our department, the effective x‐ray energy is expected to differ between the two scan types. This difference in effective energy may influence HU values and, consequently, calcification scoring. To investigate this hypothesis, ED phantoms (Model 062 M; CIRS, Norfolk, VA, USA) illustrated in Figure 1 were used to assess the relationship between HU values obtained from the two scan types. The first ED phantom (ED1), 18 cm in diameter, was constructed from soft tissue equivalent epoxy resin and contained nine rod inserts simulating adipose, breast, muscle, liver, cortical bone, and bone with hydroxyapatite (HA) density of 200, 800, 1000, 1250 mg/cc. The second ED phantom (ED2), 23 cm in diameter, was created by covering ED1 with a 2.5‐cm‐thick Superflab bolus (Radiation Products Design Inc, Albertville, MN, USA). The third ED phantom (ED3), 28 cm in diameter, was formed by applying two layers of the same bolus over ED1. The fourth ED phantom (ED4), 32 cm along the long axis and 27 cm along the short axis, combined ED1 with an additional annulus of soft tissue equivalent epoxy resin containing eight rod inserts simulating lung (inhale and exhale), adipose, breast, muscle, liver, bone with HA density of 200 and 800 mg/cc. The ED phantoms were scanned using the routine CSCT and LDCT protocol, as summarized in Table 1. During the CSCT scan, a cardiac simulator (Cardiac Trigger, Model: CTM300, Ivy Biomedical Systems, Branford, CT, USA) was used to synthesize ECG signal at a mean HR of 60 bpm. A circular region of interest (ROI) with a 2‐cm diameter was placed at the center of each rod insert in phantom images acquired with CSCT and LDCT scans to derive a calibration curve for mapping HU values from LDCT to CSCT via curve fitting. The optimal fit was determined based on the sum of squares due to error (SSE) and the coefficient of determination (*r* ^2^).

### 2.5 Calcium scoring

CSCT and LDCT before and after HU‐based calibration were transferred to an offline workstation (Aquarius iNtuition Edition ver.4.4.8, TeraRecon, Inc. USA) for CAC quantification via Agatston and volume scores. ROI containing calcified plaques in each coronary artery were manually outlined by a dedicated radiological technologist with eight years of experience, and lesion measurements were automatically calculated by the software. Calcifications with a density of ≥ 130 HU and spanning at least three contiguous pixels were considered potential coronary calcifications. The Agatston score was calculated as the sum of individual lesion scores, with each score defined as the pixel area multiplied by a weighted density factor (1: 130–199 HU, 2: 200–299 HU, 3: 300–399 HU, 4: > 399 HU). The volume score was determined by multiplying the volume of each voxel by the number of voxels exhibiting calcification. Total calcium scores were obtained by summing the scores from the right coronary artery (RCA), left anterior descending artery (LAD), left circumflex artery (LCX), and left main artery (LM). Branches were included as part of the main vessel, and the ramus intermedius was considered part of the LAD, if present.

### 2.6 Statistical analysis

For all enrolled patients, the CAC scores measured on CSCT and LDCT before and after HU‐based calibration were evaluated using correlation and Bland‐Altman analyses. Linear regression was performed, and the r^2^ was calculated to evaluate the strength of association. As for Bland‐Altman analysis, an alpha level of 0.05 was applied to determine the 95% limits of agreement (LoA). Because high HR may affect CAC scores in non‐ECG‐gated CT, patients were further stratified into four subgroups: those undergoing diastolic reconstruction with HR < 60 bpm (39 men, 10 women), 60–65 bpm (25 men, 10 women), > 65 bpm (20 men, 14 women), and those undergoing systolic reconstruction (21 men). For subgroup analysis, the CAC scores measured on CSCT and LDCT before and after HU‐based calibration were similarly evaluated using correlation and Bland‐Altman analyses. In addition, two‐sample t‐tests were performed to compare per‐artery CAC scores measured on CSCT and LDCT before and after calibration, with a significance level of *p* = 0.05.

## 3 RESULTS

### 3.1 Phantom study and HU‐based calibration

**FIGURE 2:**
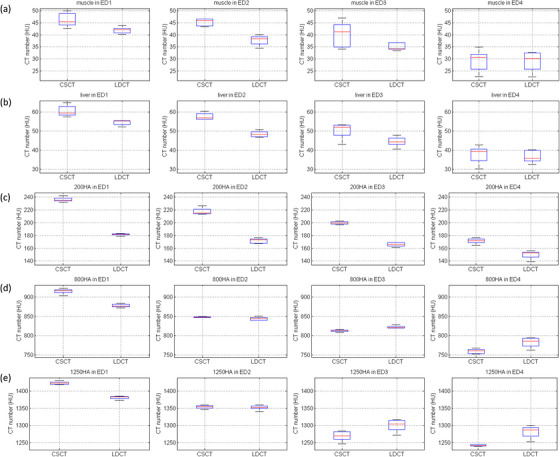
Box plots for HU values measured on CSCT and LDCT for rod inserts simulating (a) muscle, (b) liver, and bone with HA density of (c) 200, (d) 800, (e) 1250 mg/cc within ED1‐ED4 (left to right).

**FIGURE 3:**
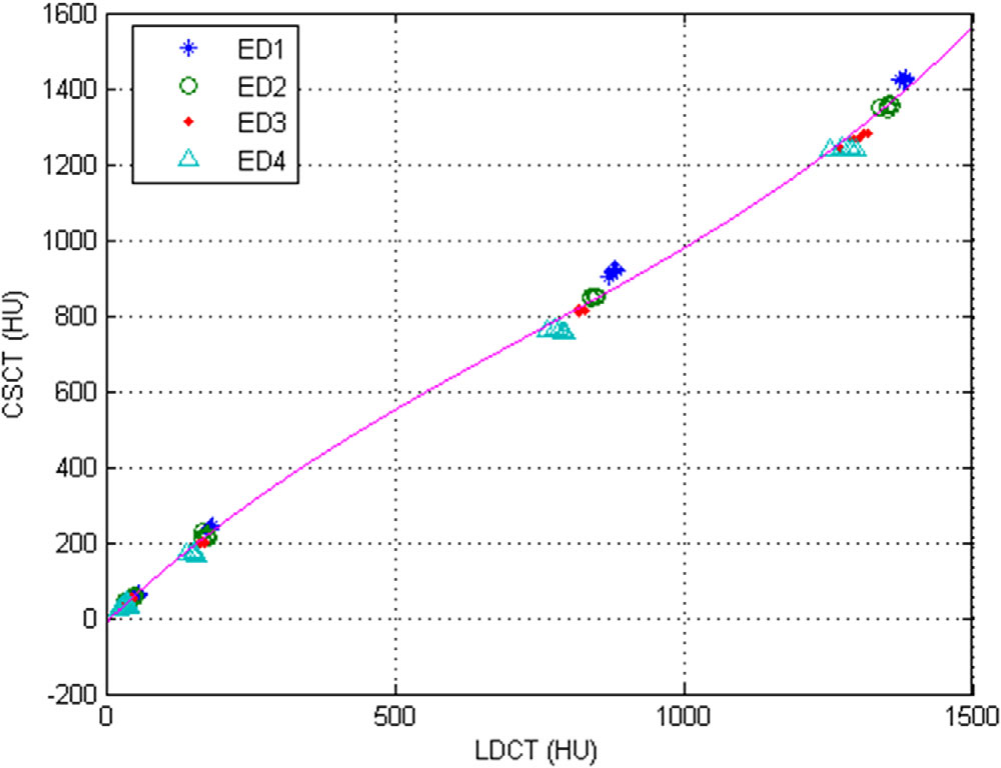
The calibration curve converting HU values in LDCT into those in CSCT.

Figure 2 shows the box plot for HU values measured from rod inserts simulating muscle, liver, and bone with HA density of 200, 800 and 1250 mg/cc. Figure 3 shows the relationship between HU values measured on LDCT and CSCT. When performing curve fitting between CSCT and LDCT, the SSE for first‐, second‐ and third‐order polynomial equations were 57480, 57070, and 33690, respectively, while the corresponding *r* ^2^ were 0.9978, 0.9978, and 0.9987. A fourth‐order polynomial equation was badly conditioned. Hence, a third‐order polynomial equation was used to generate the calibration curve for energy mapping, which was:

(1) $$\begin{array}{cc} I_{\mathrm{CSCT}}= & \;3.871\times 10^{-7}{\left(I_{\mathrm{LDCT}}\right)}^{3}-8.504\times 10^{-4}{\left(I_{\mathrm{LDCT}}\right)}^{2}\\ & +\;1.453(I_{\mathrm{LDCT}})-12.97 \end{array}$$

where I_CSCT_ and I_LDCT_ were the HU values measured on CSCT and LDCT scans, respectively.

### 3.2 Patient study and statistical analysis

#### 3.2.1 Comparison of HU values and CAC scores

**FIGURE 4:**
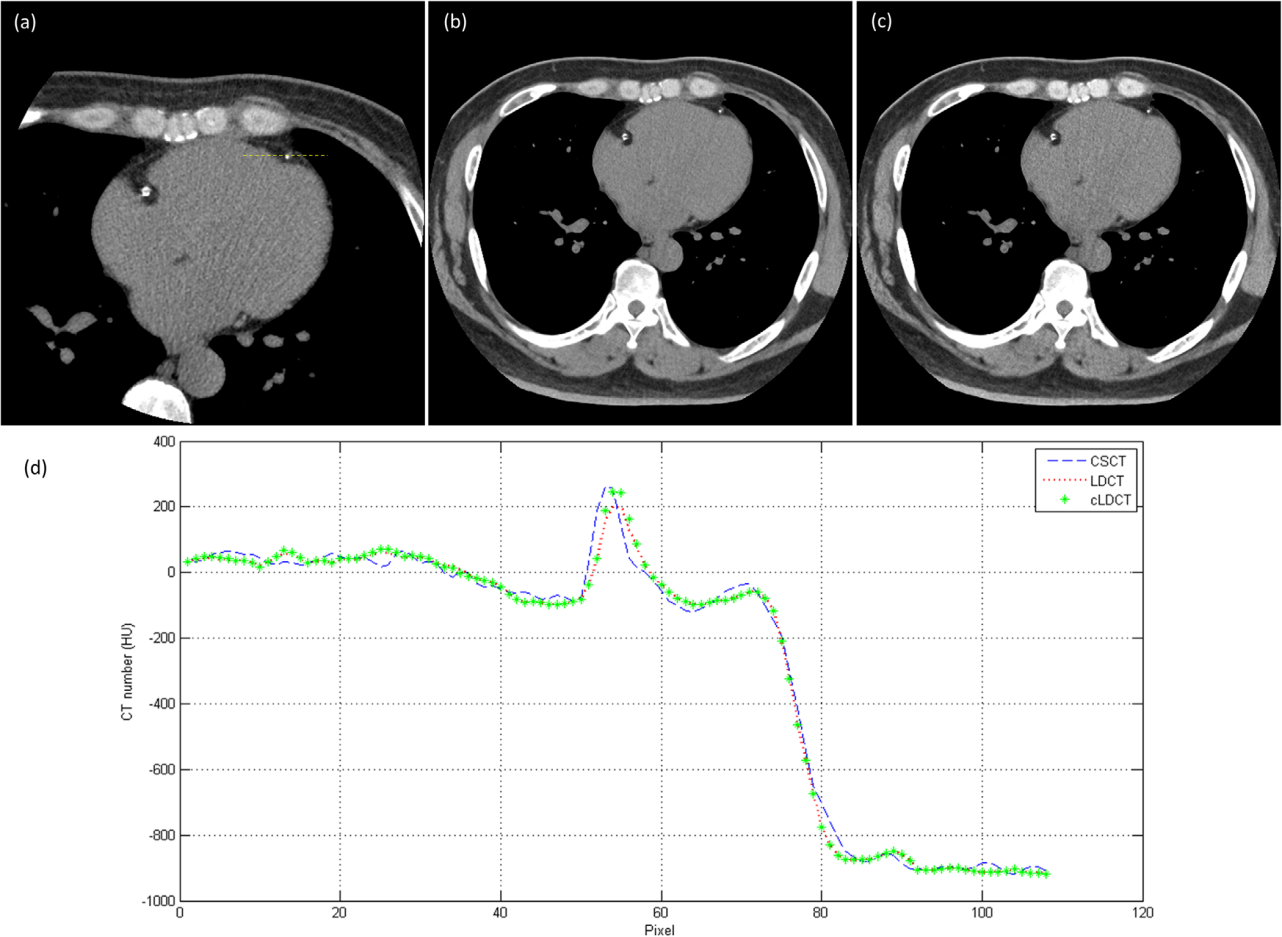
Axial images from (a) CSCT, (b) LDCT, (c) cLDCT and (d) corresponding intensity profiles across the calcification indicated by the yellow dashed line.

**FIGURE 5:**
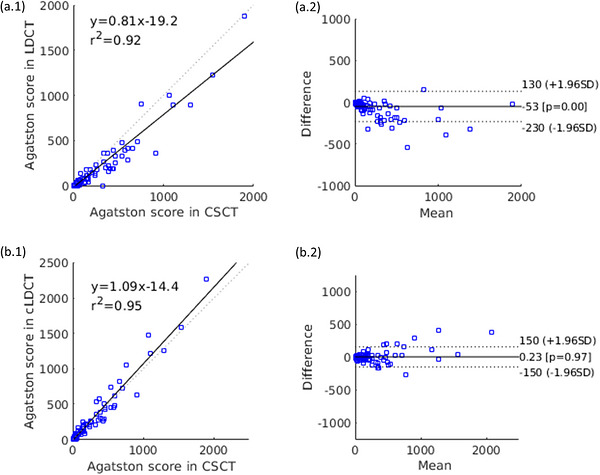
Statistical analysis of Agatston scores for all enrolled patients (*n* = 139). (a.1) Correlation plot and (a.2) Bland‐Altman plot comparing Agatston scores between CSCT and LDCT; (b.1) correlation plot and (b.2) Bland‐Altman plot comparing Agatston scores between CSCT and cLDCT.

**FIGURE 6:**
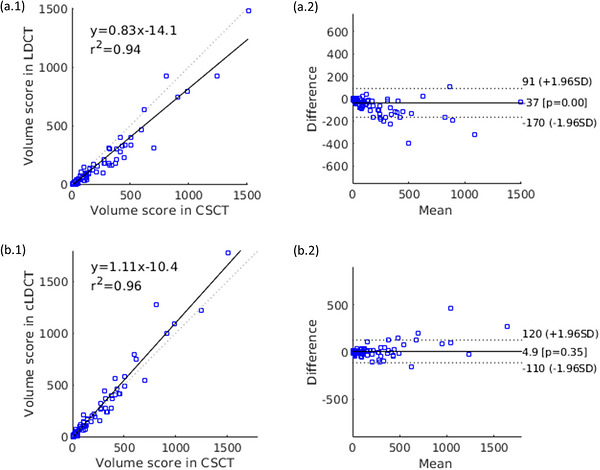
Statistical analysis of volume scores for all enrolled patients (*n* = 139). (a.1) Correlation plot and (a.2) Bland‐Altman plot comparing volume scores between CSCT and LDCT; (b.1) correlation plot and (b.2) Bland‐Altman plot comparing volume scores between CSCT and cLDCT.

Figure 4 shows axial images and intensity profiles through the yellow dashed line for CSCT, LDCT and calibrated LDCT (cLDCT) obtained using Equation (1). Figure 5(a.1) and (a.2) present the correlation and Bland‐Altman analyses of Agatston scores for all enrolled patients between CSCT and LDCT, respectively, whereas Figure 5(b.1) and (b.2) shows the corresponding analyses for Agatston scores between CSCT and cLDCT. Correlation and Bland‐Altman analyses for volume scores are presented in Figure 6. An *r* ^2^ of 0.92 was observed for Agatston scores between CSCT and LDCT, while the corresponding *r* ^2^ for volume scores was 0.94. The mean bias for the Agatston scores between CSCT and LDCT was ‐53, which was reduced to 0.23 between CSCT and cLDCT. Similarly, the mean bias for volume scores decreased from ‐37 to 4.7.

#### 3.2.2 Subgroup analysis

Table 2 summarizes the r^2^ from correlation analysis and the mean bias ± 95% LoA from Bland‐Altman analysis comparing Agatston scores between CSCT and LDCT before or after calibration for patients undergoing diastolic reconstruction with HR < 60 bpm, 60–65 bpm, > 65 bpm, and for patients undergoing systolic reconstruction. Table 3 presents the corresponding results for volume scores. The correlation plots and Bland‐Altman plots for the subgroup analyses are provided in the supplementary material S1. Across all subgroup, the r^2^ values for Agatston scores exceeded 0.86, while those for volume scores were greater than 0.92. Bland‐Altman analysis showed that the mean bias between CSCT and cLDCT was substantially lower than that observed between CSCT and LDCT.

**TABLE 2:** Subgroup analysis comparing Agatston scores between CSCT and LDCT before or after calibration.

	CSCT vs. LDCT		CSCT vs. cLDCT	
	*r* ^2^	Bias ± 95% LoA	*r* ^2^	Bias ± 95% LoA
Diastole < 60 bpm (*n* = 49)	0.96	−46 ± 137	0.96	−6 ± 91
Diastole 60–65 bpm (*n* = 35)	0.86	−59 ± 249	0.93	5 ± 184
Diastole > 65 bpm (*n* = 34)	0.95	−47 ± 132	0.96	5 ± 161
Systole (*n* = 21)	0.95	−67 ± 218	0.97	−2 ± 200

**TABLE 3:** Subgroup analysis comparing volume scores between CSCT and LDCT before or after calibration.

	CSCT vs. LDCT		CSCT vs. cLDCT	
	*r* ^2^	Bias ± 95% LoA	*r* ^2^	Bias ± 95% LoA
Diastole < 60 bpm (*n* = 49)	0.98	−33 ± 93	0.97	1 ± 72
Diastole 60–65 bpm (*n* = 35)	0.92	−51 ± 183	0.96	−2 ± 105
Diastole > 65 bpm (*n* = 34)	0.93	−27 ± 98	0.95	16 ± 168
Systole (*n* = 21)	0.97	−44 ± 133	0.99	8 ± 143

Table 4 summarizes the per‐artery Agatston scores and the total scores summed across arteries. Table 5 presents the corresponding results for the volume scores. The *p*‐values were calculated using two‐sample t‐test comparing calcium scores obtained from CSCT with those from LDCT and cLDCT. No statistically significant differences were observed in CAC scores between CSCT and cLDCT for patients undergoing diastolic reconstruction with HR ≤ 65 bpm. Although statistically significant differences were detected in the Agatston and volume scores in the LCX for patients with diastolic reconstructions at HR > 65 bpm, and the Agatston score in the RCA for patients with systolic reconstructions, no significant difference was found in the total coronary artery scores.

**TABLE 4:** Agatston scores for the four coronary arteries and the total scores summed across arteries.

		CSCT		LDCT			cLDCT		
		mean	SD	mean	SD	*P*‐value	mean	SD	*P*‐value
Diastole < 60 bpm (*n* = 49)	RCA	32.10	80.05	16.47	57.86	^*^	31.84	110.68	0.97
	LAD	64.11	126.26	43.48	83.61	^*^	56.88	102.86	0.19
	LCX	18.00	53.65	8.88	42.18	^*^	14.36	59.11	0.46
	LM	7.05	21.07	6.53	24.17	0.90	11.85	35.78	0.34
	Total	121.26	214.69	75.35	155.21	^*^	114.93	224.42	0.35
Diastole 60–65 bpm (*n* = 35)	RCA	62.32	115.90	38.66	96.16	^*^	60.72	134.31	0.84
	LAD	109.60	186.09	89.58	176.48	0.15	111.42	202.94	0.87
	LCX	22.18	73.63	12.17	48.14	^*^	22.10	68.91	0.98
	LM	11.53	36.17	9.52	33.63	0.10	16.83	59.48	0.27
	Total	205.64	326.85	146.69	266.78	^*^	211.07	346.45	0.73
Diastole > 65 bpm (*n* = 34)	RCA	72.72	179.68	54.30	165.05	^*^	85.75	248.25	0.38
	LAD	70.63	99.66	47.68	70.86	^*^	64.96	99.46	0.25
	LCX	11.27	20.29	3.10	7.94	^*^	5.21	11.12	^*^
	LM	3.58	15.75	5.75	25.60	0.64	7.70	28.15	0.31
	Total	158.20	247.11	110.84	206.17	^*^	163.62	305.48	0.70
Systole (*n* = 21)	RCA	47.51	130.41	22.15	78.86	^*^	37.62	123.25	^*^
	LAD	157.60	318.66	131.46	321.23	^*^	160.19	384.23	0.89
	LCX	33.62	62.77	17.39	36.30	^*^	31.74	57.25	0.53
	LM	17.48	50.81	18.27	64.91	0.83	25.14	76.86	0.25
	Total	256.21	476.40	189.26	432.74	^*^	254.69	537.88	0.95

*p‐value < 0.05.

**TABLE 5:** Volume scores for the four coronary arteries and the total scores summed across arteries.

		CSCT		LDCT			cLDCT		
		mean	SD	mean	SD	*P*‐value	mean	SD	*P*‐value
Diastole < 60 bpm (*n* = 49)	RCA	27.66	69.55	17.50	62.86	^*^	32.01	109.81	0.57
	LAD	49.57	99.05	35.12	69.36	^*^	45.30	85.05	0.27
	LCX	15.81	46.90	7.74	34.70	^*^	12.59	48.19	0.40
	LM	5.54	16.57	5.44	19.42	0.97	9.47	28.31	0.32
	Total	98.59	177.23	65.80	136.10	^*^	99.36	196.50	0.88
Diastole 60–65 bpm (*n* = 35)	RCA	52.56	95.93	30.98	70.83	^*^	49.14	98.04	0.47
	LAD	89.23	144.80	67.55	126.83	^*^	85.71	153.05	0.51
	LCX	18.21	60.94	11.35	41.87	0.06	19.33	60.89	0.60
	LM	9.98	31.67	9.61	34.75	0.83	13.69	48.96	0.35
	Total	169.99	266.14	119.49	201.10	^*^	167.86	266.69	0.82
Diastole > 65 bpm (*n* = 34)	RCA	55.86	136.45	48.67	151.41	0.37	73.23	212.03	0.24
	LAD	53.99	74.54	38.39	54.65	^*^	52.83	78.07	0.74
	LCX	9.33	16.55	3.03	6.89	^*^	5.48	11.37	^*^
	LM	2.65	11.93	5.21	22.59	0.53	6.45	22.99	0.21
	Total	121.84	187.40	95.29	183.87	^*^	137.98	258.33	0.28
Systole (*n* = 21)	RCA	37.57	99.16	21.61	74.40	^*^	35.95	112.87	0.68
	LAD	117.90	242.45	104.20	251.33	0.07	125.37	294.82	0.58
	LCX	28.50	53.06	15.53	32.60	^*^	28.16	50.20	0.91
	LM	16.73	51.07	15.30	52.03	0.23	19.03	61.03	0.38
	Total	200.70	375.87	156.64	348.97	^*^	208.52	432.05	0.63

* *p*‐value < 0.05.

## 4 DISCUSSION

In recent years, lung cancer screening programs have expanded globally, with increasing implementation in North America, Europe, and parts of Asia, reflecting a growing consensus on the value of LDCT screening as a public health strategy to reduce lung cancer mortality through early detection and timely intervention.^22^, ^23^ If LDCT can be employed not only for lung cancer screening but also for the simultaneous assessment of CAC, it would provide additional clinical benefit by enabling comprehensive evaluation of both pulmonary and cardiovascular health within a single examination.^24^ Although non‐gated images are subject to motion artifact and run the risk of inadequately estimating the CAC burden, several studies show that non‐gated chest CTs can produce CAC scores that correlate highly with those from dedicated scans. Kim et al. demonstrated that CAC scores derived from non‐gated chest CT show strong correlation and good agreement with those obtained from dedicated ECG‐gated calcium scoring CT.^25^ Jacobs et al. reported that CAC scores obtained from LDCT in lung cancer screening populations have been shown to be strong independent predictors of all‐cause mortality and cardiovascular events.^26^ Chamberlin et al. demonstrated that automated or AI‐based analysis of LDCT can provide reliable CAC quantification with good agreement with expert assessment.^27^ Hughes‐Austin et al. observed a strong correlation between CAC scores derived from standard 6‐mm non‐ECG‐gated chest CT and those from 3‐mm ECG‐gated scans.^28^ However, they also found that the median CAC score on the 6‐mm chest CTs was considerably lower on average than the median score on the 3‐mm ECG‐gated scans. The authors attributed this underestimation, at least in part, to the partial‐volume effect inherent in thicker slices, reducing measured peak attenuation and therefore lowering the overall score. In our study, a strong correlation between CSCT and LDCT was also observed across all enrolled patients for both the Agatston score and the volume score. However, even when identical slice thickness was used (Table 1), the regression slopes of 0.81 for the Agatston score and 0.83 for the volume score indicated that LDCT tends to yield lower calcium scores than CSCT (Figure 5 and 6). These findings suggested that factors beyond slice thickness may contribute to systematic underestimation of CAC scores on LDCT relative to CSCT.

When an x‐ray beam composed of a spectrum of photon energies passes through the silver beam‐shaping filter, lower‐energy photons are preferentially absorbed to a greater extent than the standard copper filer, resulting in an increase in the average beam energy, i.e., the beam hardening effect.^29^ Since beam hardening leads to a variation in measured HU values, energy mapping or calibration is necessary to ensure accurate quantification of CAC in LDCT images. The calibration curve developed in this study is conceptually analogous to the energy‐mapping approach employed in PET attenuation correction, wherein a 511‐keV linear attenuation map is derived from CT images through energy‐dependent calibration.^30^ Although bilinear scaling is the most common method used for energy mapping, Ay et al. reported that a quadratic polynomial function most accurately modeled their calibration curve for energy mapping in CT‐based attenuation correction. In our experience, however, a third‐order polynomial provides a significantly better fit than a second‐order function. The superior performance of the cubic function in real patient data further supports its robustness. In our opinion, the difference in optimal fitting function may be related to variations in the energy spectrum. As seen in Figure 5 and 6, the regression slopes across all enrolled patients increased to 1.09 for the Agatston score and 1.11 for the volume score after HU‐based calibration. Moreover, the Bland‐Altman plots show that the mean bias in CAC scores between CSCT and cLDCT were centered closer to zero, with narrower 95% LoA compared with those between CSCT and LDCT. A similar trend was observed in the subgroup analyses for patients undergoing diastolic reconstruction with HR ≤ 65 bpm. Although the mean bias was reduced after calibration for patients undergoing diastolic reconstruction with HR > 65 bpm, the 95% LoA increased from 132 to 161 for Agatston scores and from 98 to 168 for volume scores after calibration. Likewise, for patients undergoing systolic reconstruction, the 95% LoA for volume scores showed a slight increase from 133 to 143 after calibration. These findings were consistent with the results of the two‐sample t tests: no statistically significant differences were observed between CSCT and cLDCT in patients with HR ≤ 65 bpm, whereas significant differences between CSCT and cLDCT persisted in certain arteries among patients with HR > 65 bpm and those undergoing systolic reconstruction. Nevertheless, no significant differences were found in the total coronary artery scores between CSCT and cLDCT.

Overall, our results demonstrated that the proposed calibration method enables reliable CAC scoring on LDCT acquired for lung cancer screening using silver beam‐shaping filter and deep learning‐based reconstruction, especially for patients undergoing diastolic reconstruction with HR ≤ 65 bpm. Although its performance was slightly degraded in patients with higher HR, the method may help to identify patients undergoing LDCT who would benefit from follow‐up CSCT for further CAC evaluation. Several limitations should be acknowledged. First, the calibration curve is applicable only to LDCT scans acquired using the same protocols as those in this study. Variations in image acquisition parameters may influence HU values and, consequently, affect the accuracy of CAC scoring measured on cLDCT. Second, this study was conducted at a single center with a limited number of patients in each subgroup, which may limit generalizability. Multicenter studies with larger patient cohorts are needed to further validate these findings. Third, different beam‐shaping filters are used to reduce radiation dose in LDCT across scanners from different vendors, such as the tin filter employed in Siemens systems. Therefore, the effectiveness of the HU‐based calibration method derived from electron density phantoms should be further evaluated on CT examinations performed using scanners from multiple vendors.

## 5 CONCLUSION

An electron density phantom‐based calibration method was proposed for LDCT acquired for lung cancer screening using silver beam‐shaping filter and deep learning‐based reconstruction to align HU values and CAC scores with those from ECG‐gated CSCT. By compensating for beam hardening induced spectral differences between CSCT and LDCT, this calibration approach enables consistent HU mapping across acquisition protocols, thereby potentially enhancing the clinical utility of LDCT for simultaneous evaluation of coronary calcium burden and pulmonary pathology within a single scan.

## AUTHOR CONTRIBUTIONS

**Kuei‐Yuan Hou**: Conceptualization; methodology; software; formal analysis; writing‐review and editing; **Ching‐Ching Yang**: Conceptualization; methodology; software; validation; formal analysis; writing‐review and editing; supervision.

## CONFLICT OF INTEREST STATEMENT

The authors declare no conflicts of interest

## Supporting information


**Supporting Information**: acm270614‐sup‐0001‐SupMat.zip
