# Supplementary material for: Developing a calibration method to utilize low‐dose chest CT for assessment of coronary artery calcification score
Source: J Appl Clin Med Phys. 2026 May 12;27(5):e70614. doi: 10.1002/acm2.70614 (PMC13167253; doi:10.1002/acm2.70614)
Supplement: Supplementary file 1 — Supporting Information: acm270614‐sup‐0001‐SupMat.zip [file ACM2-27-e70614-s001.zip › 2025-08869-s01.docx]

**Developing a Calibration Method to Utilize Low-Dose Chest CT for Assessment of Coronary Artery Calcification Score**

Kuei-Yuan Hou^1,2^, Ching-Ching Yang^3,4*^

^1^ Department of Radiology, Cathay General Hospital, Taipei, Taiwan

^2^ Department of Medical Imaging and Radiological Sciences, Chung-Shan Medical University, Taichung, Taiwan

^3^ Department of Medical Imaging and Radiological Sciences, Kaohsiung Medical University, Kaohsiung, Taiwan

^4^ Department of Medical Research, Kaohsiung Medical University Hospital, Kaohsiung, Taiwan

*Corresponding author:

Ching-Ching Yang, Ph.D.

Professor

Department of Medical Imaging and Radiological Sciences, Kaohsiung Medical University,

No.100, Shin-Chuan 1st Road, Sanmin Dist., Kaohsiung, 80708, Taiwan, ROC

Tel: 886-7-312-1101

E-mail: [cyang@kmu.edu.tw](mailto:cyang@kmu.edu.tw)
