# Supplementary figures and images for: Developing a calibration method to utilize low‐dose chest CT for assessment of coronary artery calcification score
Source: J Appl Clin Med Phys. 2026 May 12;27(5):e70614. doi: 10.1002/acm2.70614 (PMC13167253; doi:10.1002/acm2.70614)

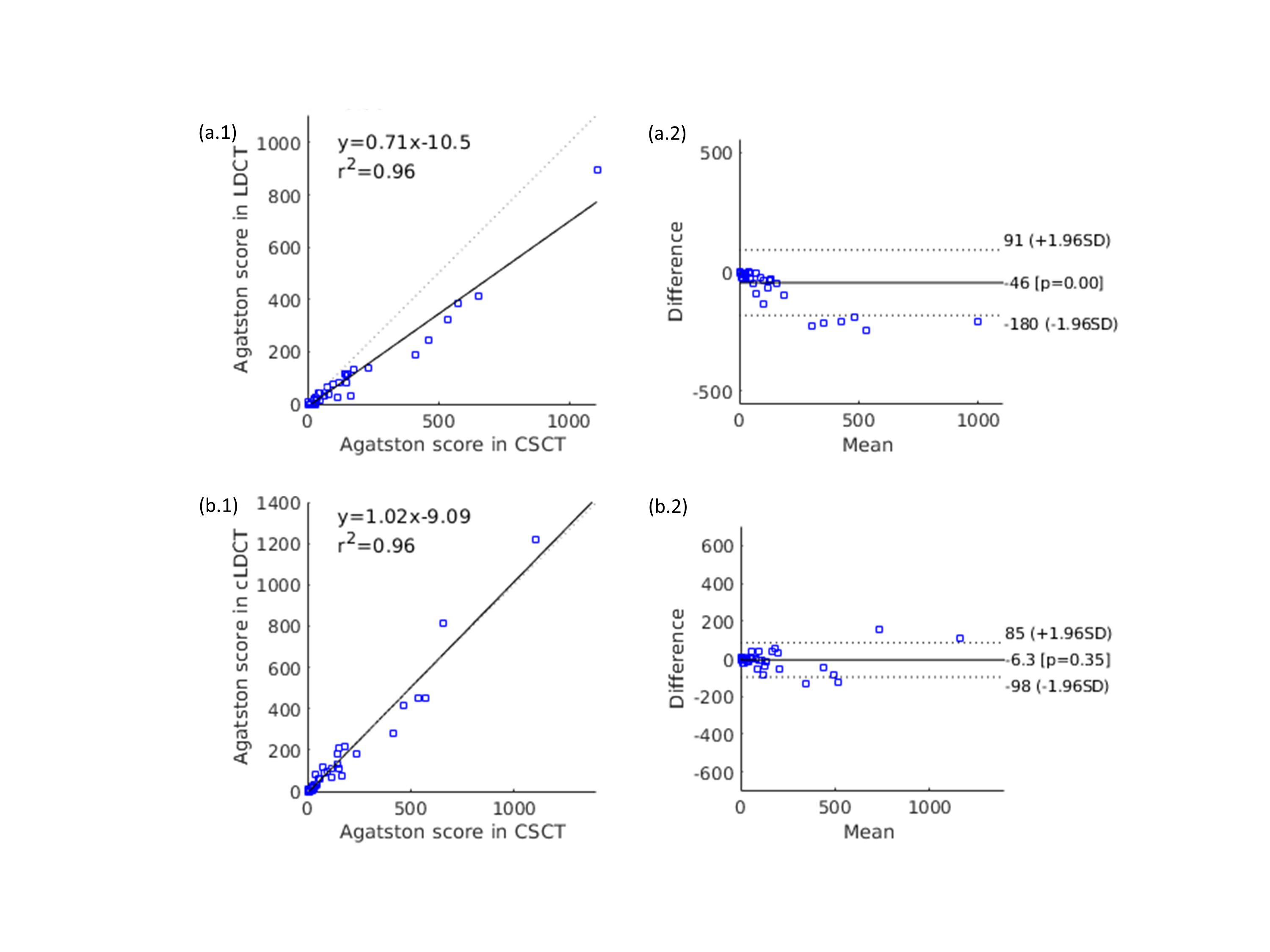

Supplement: Supplementary file 1 — Supporting Information: acm270614‐sup‐0001‐SupMat.zip [file ACM2-27-e70614-s001.zip › 2025-08869-sup-0002-SI_Figure-S01.tif]

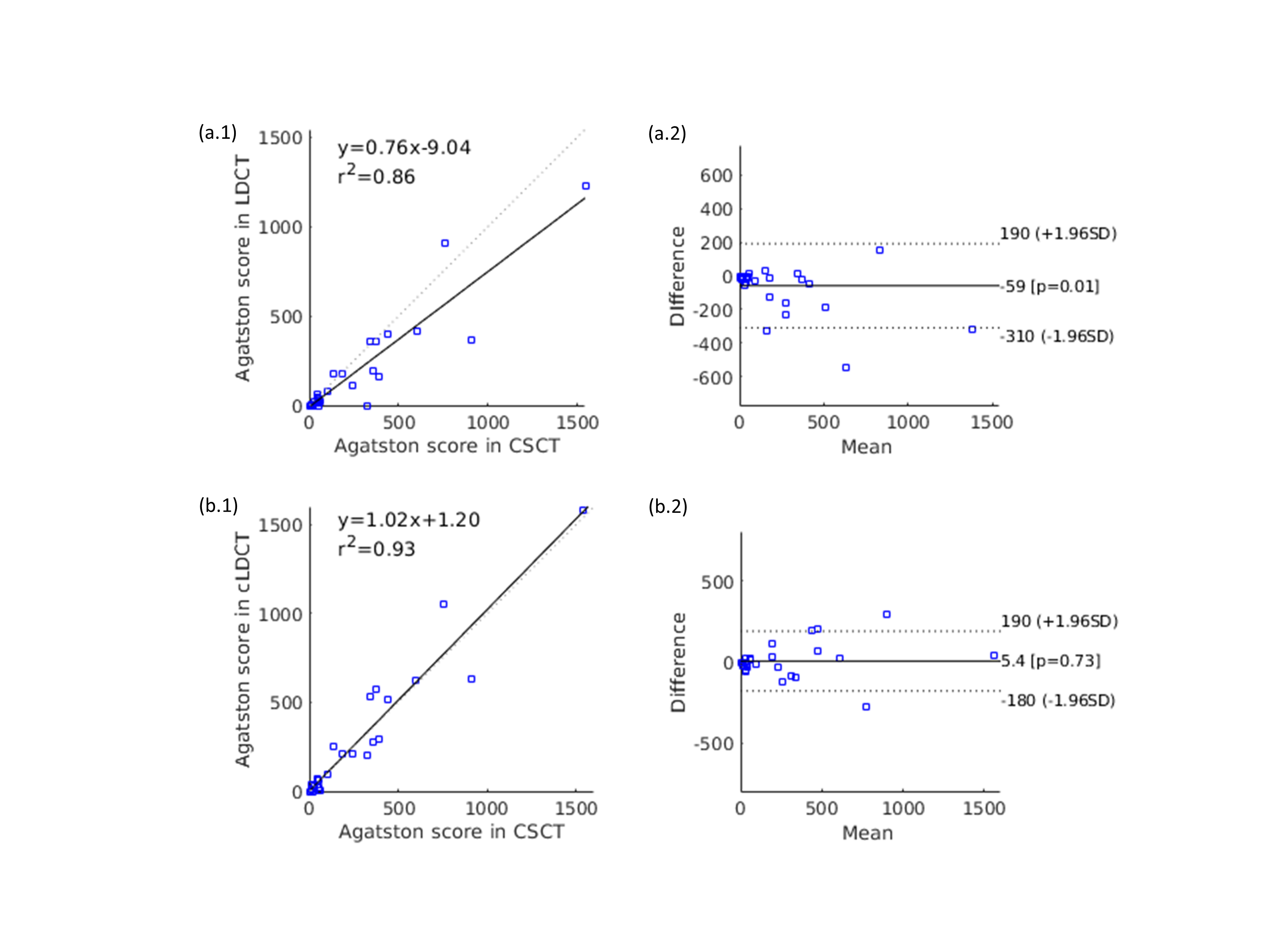

Supplement: Supplementary file 1 — Supporting Information: acm270614‐sup‐0001‐SupMat.zip [file ACM2-27-e70614-s001.zip › 2025-08869-sup-0003-SI_Figure-S02.tif]

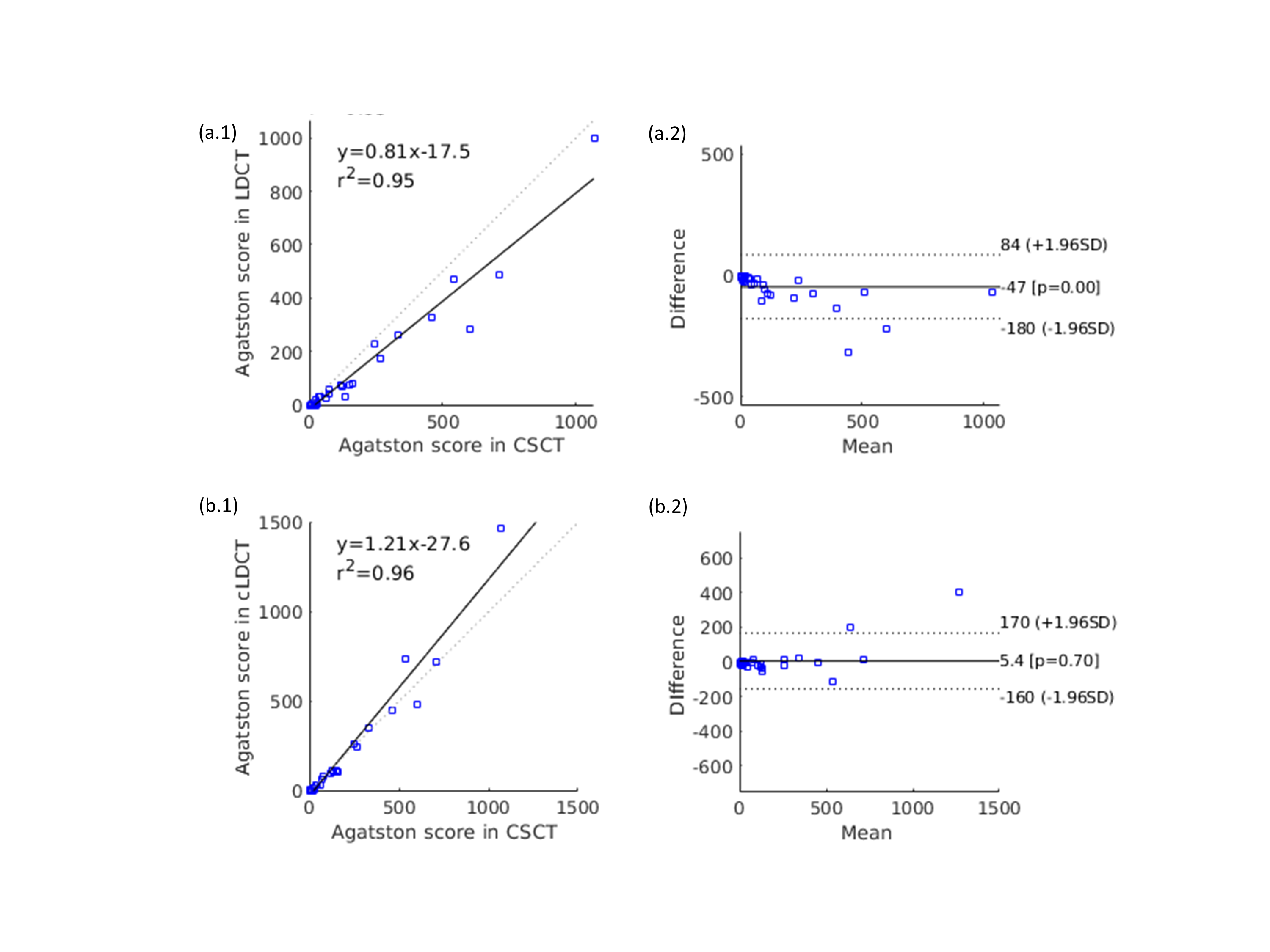

Supplement: Supplementary file 1 — Supporting Information: acm270614‐sup‐0001‐SupMat.zip [file ACM2-27-e70614-s001.zip › 2025-08869-sup-0004-SI_Figure-S03.tif]

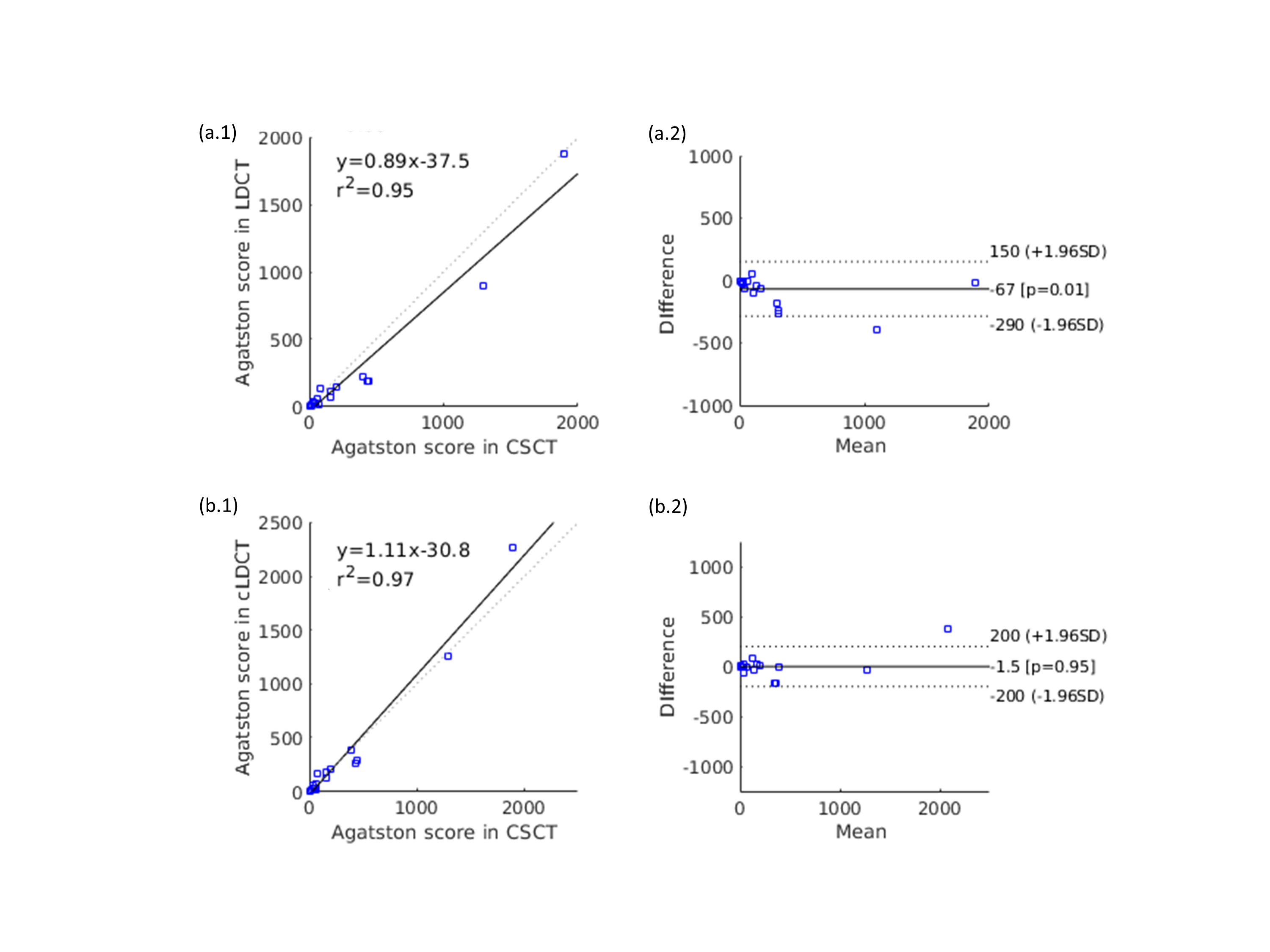

Supplement: Supplementary file 1 — Supporting Information: acm270614‐sup‐0001‐SupMat.zip [file ACM2-27-e70614-s001.zip › 2025-08869-sup-0005-SI_Figure-S04.tif]

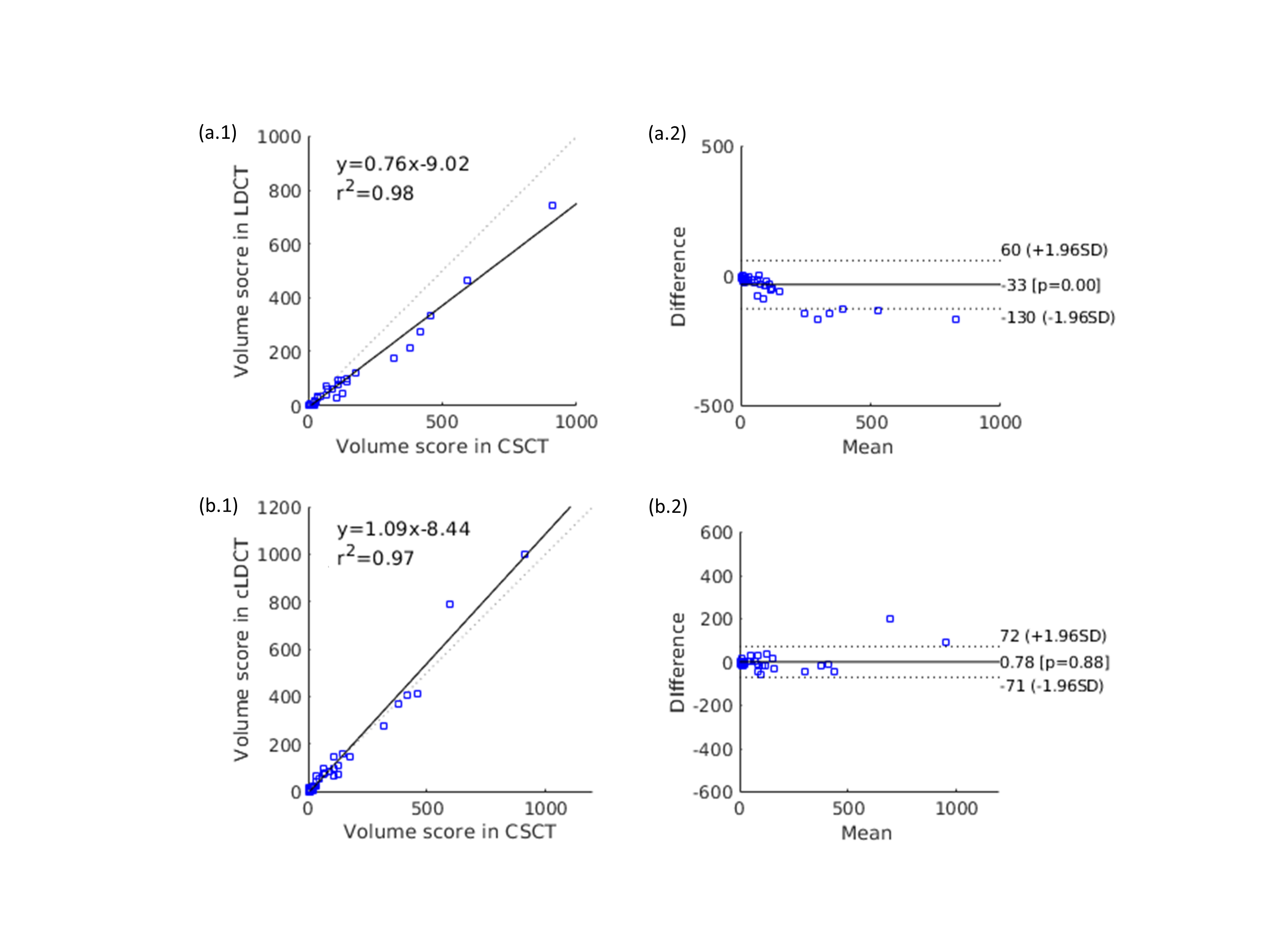

Supplement: Supplementary file 1 — Supporting Information: acm270614‐sup‐0001‐SupMat.zip [file ACM2-27-e70614-s001.zip › 2025-08869-sup-0006-SI_Figure-S05.tif]

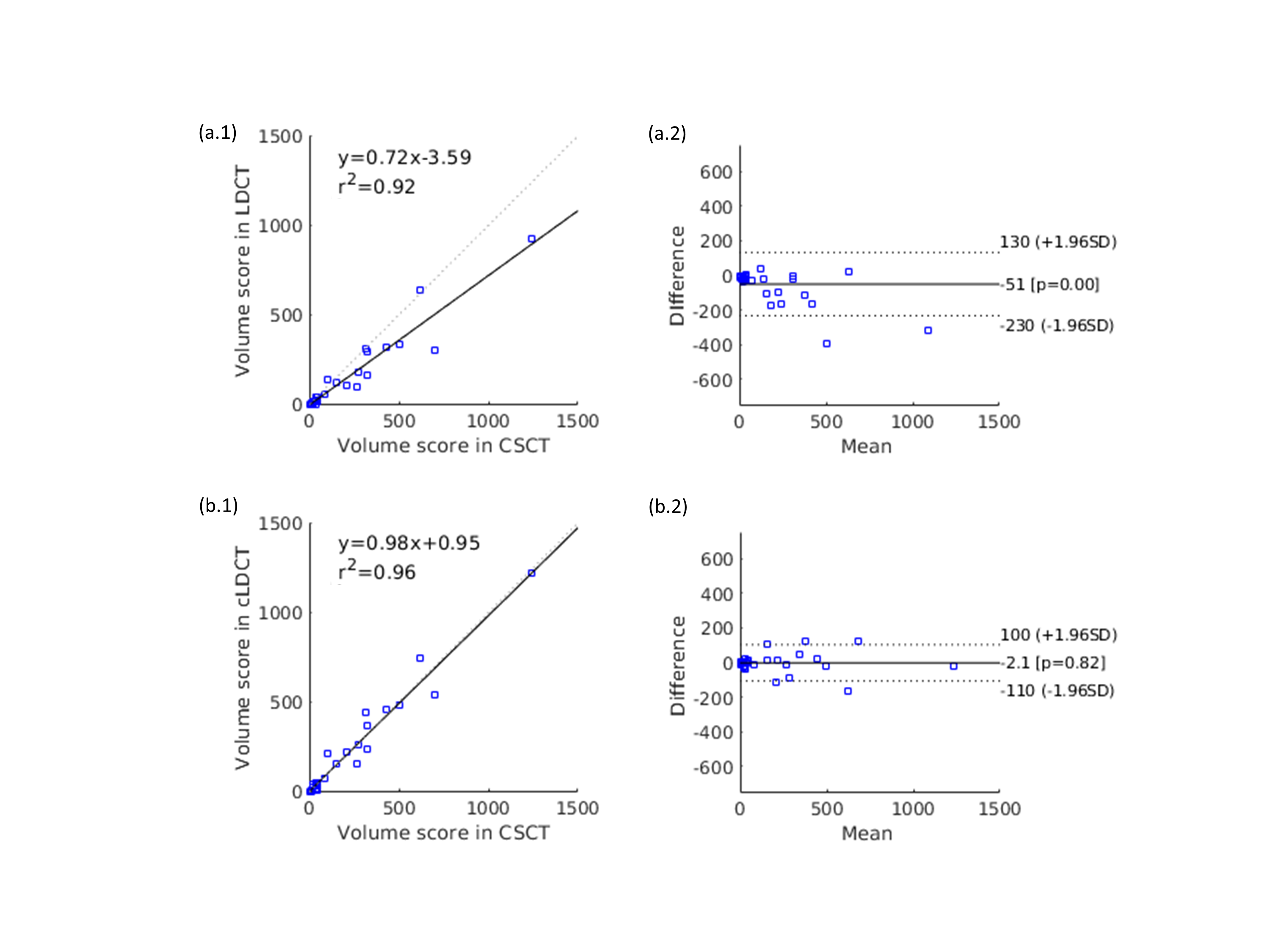

Supplement: Supplementary file 1 — Supporting Information: acm270614‐sup‐0001‐SupMat.zip [file ACM2-27-e70614-s001.zip › 2025-08869-sup-0007-SI_Figure-S06.tif]

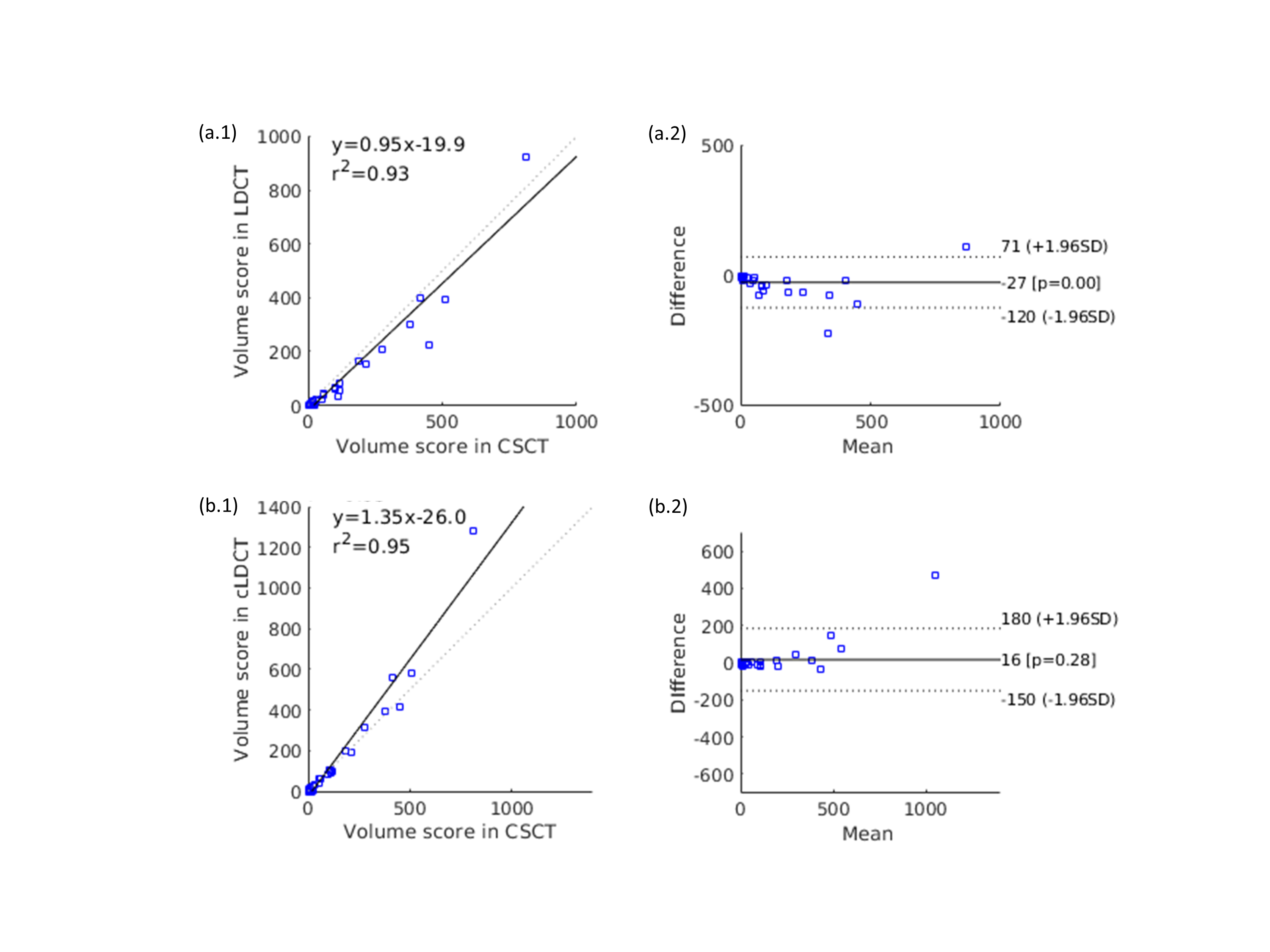

Supplement: Supplementary file 1 — Supporting Information: acm270614‐sup‐0001‐SupMat.zip [file ACM2-27-e70614-s001.zip › 2025-08869-sup-0008-SI_Figure-S07.tif]

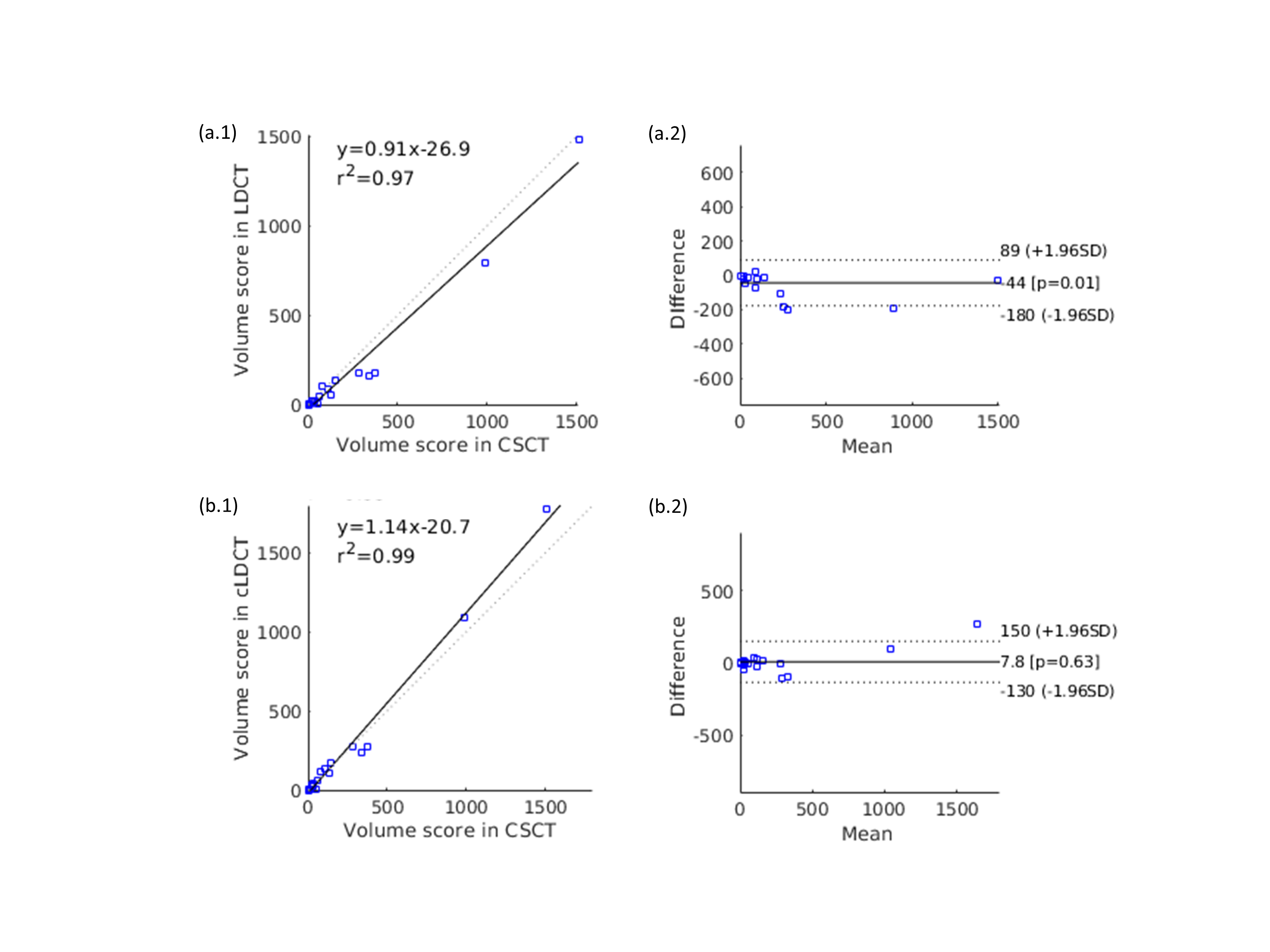

Supplement: Supplementary file 1 — Supporting Information: acm270614‐sup‐0001‐SupMat.zip [file ACM2-27-e70614-s001.zip › 2025-08869-sup-0009-SI_Figure-S08.tif]
